# Emergence of NDM-5-Producing *Escherichia coli* in a Teaching Hospital in Chongqing, China: IncF-Type Plasmids May Contribute to the Prevalence of *bla*_NDM–__5_

**DOI:** 10.3389/fmicb.2020.00334

**Published:** 2020-03-06

**Authors:** Hua Zou, Xiaojiong Jia, Hang Liu, Shuang Li, Xianan Wu, Shifeng Huang

**Affiliations:** Department of Laboratory Medicine, The First Affiliated Hospital of Chongqing Medical University, Chongqing, China

**Keywords:** carbapenem resistant, *Escherichia coli*, NDM-5, transformation, conjugation

## Abstract

The newly emerging NDM-5 confers increased antibiotic resistance and attracts extensive global attention, but the prevalence, dissemination mechanism, and clinical significance of NDM-5 among clinical *Escherichia coli* (*E. coli*) infections have not been thoroughly characterized to date. In the present study, 109 unique carbapenem-resistant *E. coli* (CR-EC) isolates were collected in Southwest China, from 2013 to 2017, among which 41 (37.61%) CR-EC isolates were identified as NDM-5-producers, with most isolates carrying the IncF-type plasmids. Molecular epidemiological studies revealed ST167 being the most common sequence type (ST). Moreover, we described the first report of a clinical CR-EC isolate co-harboring *bla*_KPC–__2_ and *bla*_NDM–__5_, which showed a higher level of resistance to carbapenems. In addition, *bla*_NDM–__5_ plasmid transformation and conjugation indicated that *bla*_NDM–__5_ itself did confer resistance to carbapenems. Complete sequencing of the *bla*_NDM–__5_-harboring IncF plasmid revealed highly conserved regions (*ble*_MBL_-*trpF*-*tat*) and some transposons around *bla*_NDM–__5_. Our findings revealed a new potential threat of NDM-5-postive CR-EC in mainland China and emphasized an urgent need to control their further spread.

## Introduction

In recent years, accumulating researches have shed light on the dissemination of carbapenem-resistant *Escherichia coli (E. coli)* (CR-EC) worldwide, mainly due to the acquisition of carbapenemase-related genes located on several mobile resistance elements (Janvier et al., 2013; Cheng et al., 2016; Giufre et al., 2018).

Among the major geographically widespread carbapenemases, *bla*_NDM_ has gained further worldwide attention for its high-level resistance to many clinically available β-lactams and the incredible horizontal transfer between different isolates. Since the first report of NDM-positive isolate in 2008 (Yong et al., 2009), 24 variants of NDM enzymes have been identified to date (Wu et al., 2019). NDM-5, which differed from NDM-1 by increased carbapenemase activity and substitutions at positions 88 (Val→Leu) and 154 (Met→Leu), was first identified in a CR-EC strain in United Kingdom (Hornsey et al., 2011).

Notably, one of the major reasons for the rapid emergence and spread of NDM-5 is its location on different incompatibility typing plasmids (Feng et al., 2018; Wang et al., 2018). Various plasmid incompatibility typing groups carrying *bla*_NDM–__5_ have been reported worldwide with the most prevalent being IncFIA/B and IncX3 in Korea (Baek et al., 2019), IncFIA and IncFIB in Egypt (Gamal et al., 2016), IncFIA and IncFK in India (Ahmad et al., 2018), and IncFII in the United States (Rojas et al., 2017). Previous studies in China have shown several sporadic cases of clinical infections linked to ST167-type *E. coli* carrying the *bla*_NDM–__5_ gene, which was mainly located on the IncX3 plasmid (Chen et al., 2014; Yang et al., 2014; Li et al., 2018; Sun et al., 2019). Unexpectedly, our initial study revealed that the epidemic incompatibility type of NDM-5 plasmid in our region is IncF, representing a new potential threat of carbapenem resistance *Enterobacteriaceae* (CRE) in China.

More importantly, the coproduction of NDM-5 and other carbapenemases in a single isolate is worthy of special concern, since it has been demonstrated to confer a higher-level resistance to carbapenems (Gamal et al., 2016; Rojas et al., 2017). To the best of our knowledge, this is the first report of clinical *E. coli* strains simultaneously producing NDM-5 and KPC-2 carbapenemases.

Therefore, the present study was initiated: (i) to describe the prevalence of clinical CR-EC isolates collected successively for approximately 5 years, (ii) to identify the resistance mechanisms among these CR-EC strains, and (iii) to explore the genetic context of *bla*_NDM–__5_ to further elucidate the mechanisms involved in antibiotic resistance gene transferring.

## Materials and Methods

### Bacterial Strains

This retrospective study was performed in the First Affiliated Hospital of Chongqing Medical University and associated two branch hospitals in Southwestern China. 109 non-repetitive nosocomial CR-EC strains were collected between 2013 and 2017. All the isolates were identified at the species level by the VITEK MS (bioMérieux, Hazelwood, MO, United States) automated system, and routine antimicrobial susceptibility testing was performed by using the VITEK2 compact (bioMérieux, Inc., Durham, NC, United States) system. According to the breakpoint recommendations by the Clinical and Laboratory Standards Institute, 2017 (CLSI-2017), isolates which were non-susceptible to at least one of the carbapenems by the broth microdilution method, with the criteria of minimum inhibitory concentration (MIC) of ≥2 μg/mL for ertapenem (ETP), ≥4 μg/mL for imipenem (IPM), or ≥4 μg/mL for meropenem (MEM), were included in the study.

### Antibiotics and *in vitro* Susceptibility Testing

All the isolates were tested for antibiotic susceptibilities to ceftazidime (CAZ), ceftriaxone (CRO), cefepime (FEP), gentamicin (GM), tobramycin (TOB), ciprofloxacin (CIP), and levofloxacin (LEV) by using AST GN13 cards on the VITEK2 compact system. MICs for ETP, IPM, MEM, colistin (CS), Aztreonam (ATM), and tigecycline (TGC) were determined using the broth microdilution method. MIC results were interpreted according to CLSI-2017 breakpoint recommendations. Non-carbapenemase-producing *E. coli* strain ATCC 25922 was served as a quality-control strain.

### DNA Amplification and Analysis

Total DNA was extracted by boiling centrifugation method (Green and Sambrook, 2017). Briefly, single colonies were picked from overnight culture of each *E. coli* isolate, resuspended in 200 μl of sterile distilled water, and boiled at 100°C for 10 min. After centrifugation at 15,000 × *g* for 15 min, supernatants were collected and stored at −20 C (Rahman et al., 2014). The potential presence of carbapenemase genes, including *bla*_KPC_, *bla*_NDM_, *bla*_VIM_, *bla*_IMP_, *bla*_OXA__–__23_, *bla*_OXA__–__24_, and *bla*_OXA__–__48_, were detected by polymerase chain reaction (PCR),and all the variants of these carbapenemase genes were confirmed by sequencing (Wang et al., 2018). Furthermore, ESBL genes, such as *bla*_CTXM_, *bla*_TME_, *bla*_SHV_, and *bla*_OXA–__1_, AmpC genes, such as *bla*_ACC_, *bla*_FOX_, *bla*_MOX_, *bla*_DHA_, *bla*_CIT_, and *bla*_EBC_, porin genes, such as *ompF* and *ompC* were also determined by using primers as described previously (Supplementary Table S1). All the amplified PCR products were gel purified and sequenced by the Sanger method, while the plasmids were sequenced by the next-generation high throughput sequencing on the Illumina Hiseq 2000 (Illumina Inc., San Diego, CA, United States) platform.

### Conjugation, Transformation, and Plasmid Analysis

To assess whether the carbapenemase-producing genes were located on the plasmids, conjugation with *E. coli* EC600 and transformation with *E. coli* DH5α were repeatedly conducted. For conjugative assays, the rifampin-resistant *E. coli* EC600 strain was use as the recipient, and the transconjugants were selected on Mueller-Hinton agar plates supplemented with a combination of 32 μg/ml ampicillin and 512 μg/ml rifampin. For transformation assays, the transformants were selected on Mueller-Hinton agar plates containing 2 μg/ml MEM (Jia et al., 2018). Both the transconjugants and transformants were tested for antimicrobial susceptibilities by the VITEK2 compact system, and MICs for ETP, IPM, MEM, colistin (CS), and tigecycline (TGC) were further determined by the broth microdilution method. The presence of resistance determinants were confirmed by PCR.

Plasmids carrying *bla*_NDM_ were extracted from transconjugants using a QIAGEN Plasmid Mini Kit (QIAGEN, Germany) according to the manufacturer’s instructions. To investigate the genetic context around the *bla*_NDM–__5_ gene, we first chose the plasmid pNDM5-1001 which was extracted from transconjugant and harbored a *bla*_NDM–__5_ gene for sequencing. The whole plasmid sequence was obtained on the HiSeq 2000 platform (Illumina Inc., San Diego, CA, United States) generating 400 bp paired-end reads. Then, the derived reads were trimmed and assembled using SOAP *de novo* v2.04^1^, and gaps were closed through PCR and Sanger sequencing. Sequence analysis was done by both the ORF Finder^2^ and the BLAST functions^3^. The genomes of the plasmids were annotated by GeneMarkS^4^. The circular map of the pNDM5-1001 plasmid was generated using the Snap Gene server^5^.

A comparison of pNDM5-1001 and four other related plasmids pGUE_NDM (accession number JQ364967.1), pNDM5-020007 (accession number CP025626.1), pNDM-5-IT (accession number MG649062.1), and pNDM-BJ01 (accession number JQ001791.1) was performed with Mauve^6^.

Incompatibility typing of the *bla*_NDM_ plasmid was performed by PCR-based replicon typing (Carattoli et al., 2005).

### Genetic Environments of *bla*_NDM_-Carrying Plasmids

The genetic environment of *bla*_NDM_ was established by PCR as described previously (Zhang et al., 2016). Primers were designed based on the reported *bla*_NDM_ flanking sequences to determine the genetic background of the *bla*_NDM_-harboring strains (Supplementary Tables S2, S3). PCR experiments were performed using the same thermocycling conditions for all strains as follows: one cycle of 94°C for 5 min; followed by 35 cycles of 94°C for 35 s, 56°C for 45 s, and 72°C for 1 min; and a final cycle at 72°C for 10 min. The amplified products were sequenced and compared with the similar sequences deposited in the BLAST database, and the DNA sequences obtained were compared with those available in the NCBI GenBank database.

### Multi-Locus Sequence Typing (MLST) and Pulsed-Field Gel Electrophoresis (PFGE)

Multi-locus sequence typing (MLST) was performed by the amplifications of the internal fragments of seven housekeeping genes of carbapenemase-producing *E. coli* isolates (*adk*, *fumC*, *icd*, *purA*, *gyrB*, *recA*, and *mdh*) according to the database^7^. The clonal relationships of the isolates harboring the *bla*_NDM–__5_ gene were further determined by pulsed-field gel electrophoresis (PFGE). Briefly, genomic DNA of the *bla*_NDM–__5_-positive *E. coli* isolates was prepared in agarose blocks and digested with restriction enzyme *Xba*I. DNA fragments were separated using a CHEF II D-Mapper XA PFGE system (Bio-Rad, Hercules, California, CA, United States) with running conditions as described previously (Jain et al., 2018).

### Ethical Considerations

The data and samples analyzed in the present study were obtained in accordance with the standards and approved by the Chongqing Medical University Institutional Review Board and Biomedical Ethics Committee. For this study, samples were collected at the microbiology laboratory of our hospital, with no contact with the patients. This study was retrospective and there was no patient identification performed during data collection. Therefore, the ethics committee determined that informed consent was not required.

### Statistical Analysis

All analyses were performed using SPSS v.25.0 software (SPSS Inc., Chicago, IL, United States). Univariate analyses were performed separately for each of the variables. All variables with a *P* value of ≤0.05 in the univariate analyses were considered for inclusion in the multivariate logistic regression model. The odds ratio (OR) and 95% confidence interval (CI) were calculated to evaluate the strength of any association. Categorical variables were calculated using a chi-square test or Fisher’s exact test as appropriate. Continuous variables were calculated using Student *t* test (normally distributed variables) and Wilcoxon rank-sum test (non-normally distributed variables) as appropriate. For all calculations, statistical significance was defined at *P* < 0.05 for 2-tailed tests.

## Results

### General Characteristics and Antimicrobial Susceptibility of CR-EC Isolates

As shown in Table 1, a total of 109 non-repetitive CR-EC strains were isolated from 2013 to 2017 in our hospitals. These non-duplicated isolates were mainly isolated from urine, followed by the respiratory tract samples, drainage, secretion, blood, and other sources including bile and ascites. For the antimicrobial susceptibility of these isolates, our study showed the highest non-susceptible rate to ETP (97.3%), with 57.8% and 55.9% non-susceptible rates to imipenem and meropenem, respectively. Almost all the CR-EC isolates showed resistances to cephalosporins, especially to CRO, CAZ and FEP. Although NDM does not normally show activity against the monobactams such as aztreonam, CR-EC isolates showed high resistances to aztreonam, about 88.9%, probably due to the high prevalence of *bla*_CTX–M_ among these isolates (Table 1). Moreover, over half of the CR-EC strains exhibited resistance to aminoglycoside and gentamycin. However, these CR-EC isolates were mostly susceptible to tigecycline and colistin. Compared with carbapenemase-negative isolates, our research revealed that a significantly greater proportion of carbapenemase-positive *E. coli* isolates were resistant to most antibiotics, including ETP, imipenem, meropenem, FEP, CIP, and LEV. Notably, 86.2% (94/109) of the CR-EC isolates were identified to be multi-drug resistant (MDR) as they were resistant to three or more classes of antimicrobial agents.

**TABLE 1:** Antimicrobial susceptibility of CR-EC isolates with or without carbapenemases.

Antimicrobial agents	Total (*N* = 109)	Carbapenemase positive (*N* = 50)				Carbapenemase negative (*N* = 59)				*P* value
	R (%)	R	MIC_50_	MIC_90_	Range	R	MIC_50_	MIC_90_	Range	
Ertapenem	106 (97.3)	50 (100.0)	32	256	2–512	56 (94.9)	4	64	1–512	< 0.001
Imipenem	63 (57.8)	50 (100.0)	32	128	4–512	13 (22.0)	1	8	0.25–32	< 0.001
Meropenem	61 (55.9)	48 (96.0)	8	54	0.25–256	13 (22.0)	1	8	0.25–8	< 0.001
Ceftriaxone	109 (100.0)	50 (100.0)	128	512	64–512	59 (100.0)	64	128	16–512	–
Ceftazidime	102 (93.6)	50 (100.0)	128	512	64–512	52 (88.2)	64	128	1–256	< 0.001
Cefepime	101 (92.7)	49 (98.0)	128	512	8–512	52 (88.1)	64	128	1–256	< 0.001
Aztreonam	97 (88.9)	48 (96.0)	128	512	8–512	49 (83.0)	64	256	2–256	< 0.001
Ciprofloxacin	85 (77.9)	41 (82.0)	16	32	0.25–32	44 (74.6)	4	8	0.25–16	< 0.001
Levofloxacin	76 (69.7)	37 (74.0)	16	64	0.25–64	39 (66.1)	8	8	0.25–32	< 0.001
Gentamycin	70 (64.2)	34 (68.0)	32	128	1–512	36 (61.0)	16	16	1–32	0.02
Tobramycin	51 (46.8)	24 (48.0)	8	128	1–512	27 (45.8)	8	16	1–32	0.735
Colistin	22 (20.2)	12 (24.0)	1	4	1–8	10 (16.9)	1	8	1–8	0.988
Tigecycline	0 (0.00)	0 (0.00)	1	4	0.25–4	0 (0.0)	0.25	1	0.25–2	–

*Data are number resistant (% of resistance rates). *P*-value for comparisons of the resistance rates of carbapenemase-positive and carbapenemase-negative groups. Bold face indicates values that are significant (*P* < 0.05). R, resistance.*

### Genotypic Distribution of the CR-EC Isolates

Among the 109 CR-EC isolates, 50 (45.9%) strains were demonstrated to be carbapenemase-producers, and most notably, all the 50 strains were positive for NDM, among which 41 (82.0%) of them were NDM-5-positive, 8 (16.0%) were NDM-1-positive, and 1 (2.0%) was NDM-9-positive. Notably, there was one isolate co-harboring *bla*_NDM–__5_ and *bla*_KPC–__2_. In addition to the production of carbapenemases, 76.2% (83/109) of the isolates were demonstrated to be ESBLs-positive, and *bla*_CTX–M_ was the most prevalent ESBLs gene in the CR-EC isolates, with *bla*_CTX–M–__1_ being the most prevalent (42.2%, 46/109) subtype, followed by *bla*_CTX–M–__9_ (22.9%, 25/109) and *bla*_CTX–M–__15_ (21.1%, 23/109) subtypes. Notably, all the 50 NDM-positive isolates were demonstrated to be co-harboring ESBLs. Besides *bla*_CTX–M_, *bla*_TEM_ (exclusively *bla*_TEM–__1_) also showed a high prevalence (32.1%, 35/109). However, no AmpC positive strains were identified. In addition, loss of expression of OmpC and OmpF were not common in CR-EC isolates, only accounting for 24.8% (27/109) and 19.3% (21/109) of these isolates. Notably, 45.9% (50/109) of the isolates were demonstrated to be co-harboring carbapenemases and ESBLs; and 11.0% (12/109) of the isolates co-produced carbapenemases and ESBLs with OMPs loss (Table 2). Most of the patients carrying NDM-producers were from ICU (28%, 14/50), the hepatological surgery department (12%, 6/50), and the gastrointestinal surgery department (8%, 4/50). Patients who didn’t carry NDM-producers were mostly from the hepatological surgery department (23.7%, 14/59), the urinary surgery department (11.86%, 7/59), and the gastrointestinal surgery department (10.16%, 6/59). It was interesting that isolates carrying the *bla*_NDM–__5_ gene were mostly from sputum (10/50, 20.0%), urine (8/50, 16.0%), and blood (6/50, 12.0%), nevertheless, NDM negative isolates were mostly from urine (20/59, 33.8%), bile (7/59, 11.8%), and sputum (7/59, 11.8%).

**TABLE 2:** Presence of antibiotic resistance genes in clinical carbapenemase-positive and carbapenemase-negative CR-EC isolates.

Carbapenem resistance mechanisms		Total (109)	Carbapenemase positive (50)	Carbapenemase negative (59)
Carbapenemase type(s)		50(45.9%)		
NDM- positive	NDM-1	8(7.3%)	8(16.0%)	NA
	NDM-5	41(37.6%)	41(82.0%)	
	NDM-9	1(0.9%)	1(2.0%)	
KPC- positive	KPC-2	1(0.9%)	1(2.0%)	NA
NDM-5, KPC-2		1(0.9%)	1(2.0%)	NA
ESBL type(s)		83(76.2%)		
CTXM- positive	CTXM-1	46(42.2%)	24(48.0%)	22 (37.3%)
	CTXM-9	25(22.9%)	16(32.0%)	9 (15.3%)
	CTXM-15	23(21.1%)	16(32.0%)	7 (11.9%)
TEM- positive	TEM-1	35(32.1%)	29(58.0%)	6 (10.2%)
OXA-1-positive	OXA-1	23(21.1%)	8(16.0%)	15 (25.4%)
OMPs loss		32(29.4%)		
OMPs	OmpC	27(24.8%)	12(24.0%)	15 (25.4%)
	OmpF	21(19.3%)	11(22.0%)	10 (16.9%)
	OmpC, OmpF	16(14.7%)	4(8.0%)	12 (20.3%)
Carbapenemase, ESBL type		50(45.9%)	50(100.0%)	NA
Carbapenemase, ESBL type, OMPs loss		12(11.0%)	12(24.0%)	NA
Carbapenemase, OMPs loss		12(11.0%)	12(24.0%)	NA
ESBL type, OMPs loss		27(24.8%)	12(24.0%)	15 (25.4%)

*NA, not applicable.*

### Characteristics of the Isolated CR-EC Strains Carrying *bla*_NDM–__5_

**FIGURE 1:**
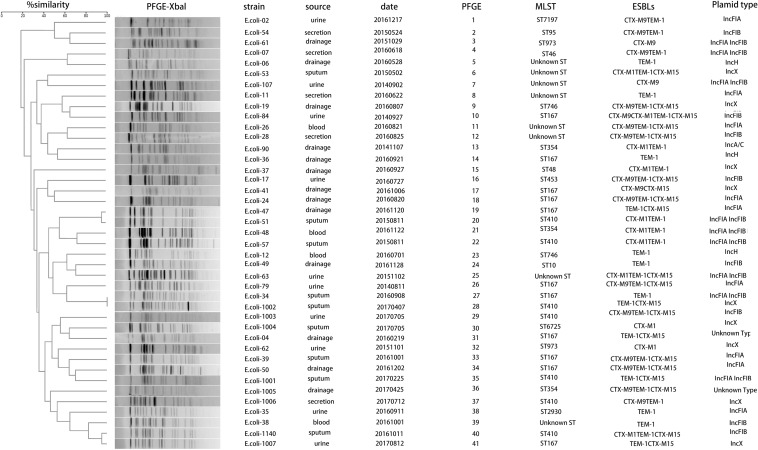
Dendrogram analysis and molecular epidemiology of NDM-5 positive *Escherichia coli* isolates. The dendrogram is based on the similarity of PFGE patterns from 41 *bla*_NDM–__5_ positive clinical *E. coli* isolates. The right columns illustrate results of source, date, PFGE, MLST, ESBLs, and plasmid types.

The *bla*_NDM–__5_-carrying CR-EC isolates were found to be more resistant to carbapenems than CR-EC isolates carrying other *bla*_NDM_ subtypes. While PCR assays demonstrated that *bla*_NDM–__5_ was successfully transferred by both conjugation and transformation experiments in all the CR-EC isolates carrying the *bla*_NDM–__5_ gene, multiple attempts to co-transfer or co-conjugate *bla*_KPC–__2_ and *bla*_NDM–__5_ failed, and no transconjugants or transformants co-harboring *bla*_KPC–__2_ and *bla*_NDM–__5_ were detected. Notably, all of the transconjugants and transformants acquired multi-drug resistance phenotypes similar to those of the donors except for reduced MICs to carbapenems and cephalosporins, and still remained susceptible to colistin and tigecycline. According to the plasmid replicon typing results, plasmid types of the transconjugants and transformants harboring *bla*_NDM_ were IncF (33/50, 66.0%), IncX (11/50, 22.0%), IncH (3/50, 6.0%), and IncA/C (1/50, 2.0%). For chromosomal characteristics, the most prevalent sequence types (STs) was ST167 (12/50, 24.0%), followed by ST410 (5/50, 10.0%) and ST354 (3/50, 6.0%). Some rare ST types, including ST973 and ST746, were also witnessed, only accounting for 4.0% of the isolates, respectively. Notably, 16.0% of the CR-EC isolates still remain unknown ST types. PFGE was performed on all the 41 *bla*_NDM–__5_-positive isolates, and all but 2 (*E. coli*-34 and *E. coli*-1002) of these strains showed a different PFGE band pattern (Table 3 and Figure 1).

**TABLE 3:** Profiles of plasmids and corresponding average carbapenem MICs in 50 *bla*_NDM_-positive *E. coli* clinical isolates.

Characteristics		NDM-5	NDM-1	NDM-9
Average MICs (μg/ml)	ETP	84.79	31.75	8
	IPM	55.10	31	8
	MEM	27.67	8.13	4
MLST		ST167 (10)	ST167 (1)	ST167 (1)
		ST410 (5)	ST361 (1)	
		ST354 (3)	ST4538 (1)	
		ST973 (2)	ST453 (1)	
		ST746 (2)	Unknown ST (4)	
		Unknown ST (8) Other types (11)		
Replicon type		IncF (26)	IncF (6)	IncF (1)
		IncX (9)	IncX (2)	
		IncA/C (1)		
		IncH (3) UnknownType (2)		

*ETP, ertapenem; IPM, imipenem; MEM, meropenem.*

### Sequence Analysis of Plasmid Containing *bla*_NDM–__5_

**FIGURE 2:**
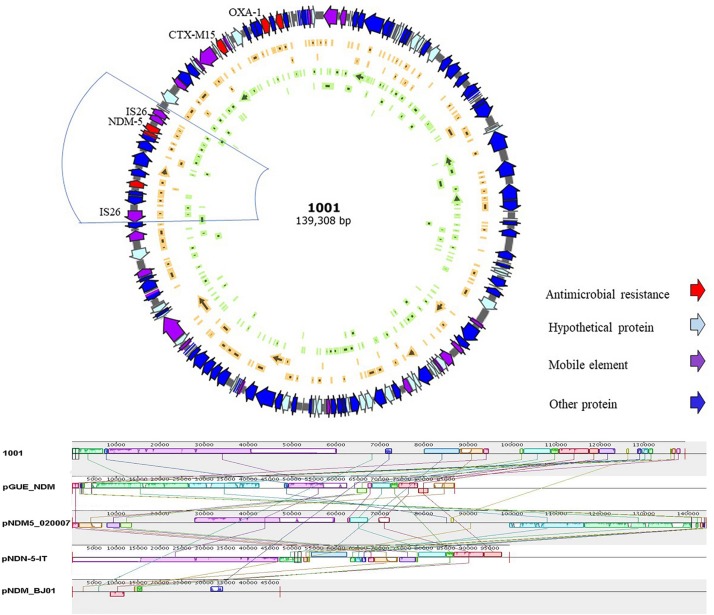
Schematic map of plasmid pNDM-5-1001 and comparative analysis of plasmid pNDM-5-1001 with other three *bla*_NDM–__5_-carrying plasmids and pNDM-BJ01. Reference sequences: pNDM-BJ01 from an *Acinetobacter lwoffii* strain (GenBank accession No. JQ001791), pNDM-5-IT from an *E. coli* strain (GenBank accession No. MG649062.1), pGUE_NDM from an *E. coli* strain (GenBank accession number JQ364967.1), and pNDM5-020007 from an *E. coli* strain (GenBank accession number CP025626.1).

High throughput sequencing analysis was conducted on *E. coli*-1001, a strain co-harboring *bla*_KPC–__2_ and *bla*_NDM–_*_5_* genes. The 139-kb pNDM-5-1001 plasmid was identified to be of the IncF replicon type: containing FIA and FIB replicons, and also co-harboring *bla*_CTX–M__15_, *bla*_OXA–__1_, aminoglycoside resistance genes [*aac(6’)-I*, *aac(6’)-II*], and tetracycline resistance gene (*tet*). Within the pNDM-5-1001 plasmid, *bla*_NDM–__5_ was bracketed by two insertion sequences IS26, containing the ISCR1 element and some other resistance genes (Figure 2). Further sequence alignments based on BLAST revealed that the plasmid sequence showed the most similar nucleotide sequences to those of the following previously reported plasmids: pGUE-NDM (accession number JQ364967.1) from an *E. coli* strain, pNDM5-020007 (accession number CP025626.1) from an *E. coli* strain, and pNDM-5-IT (GenBank accession No. MG649062.1) from an *E. coli* strain, but less similar to pNDM-BJ01 (GenBank accession No. JQ001791) from an *Acinetobacter lwoffii* (*A. lwoffii*) strain, which is the first NDM-expressing plasmid known to have been fully sequenced in China. The identical regions, including the replicon, stabilization elements, toxin-antitoxin systems (*sopA* and *sopB*) and virulence factors (*arcA*, *arcB*, *arc*, and *arcD*), were highly conserved (Figure 2).

**FIGURE 3:**
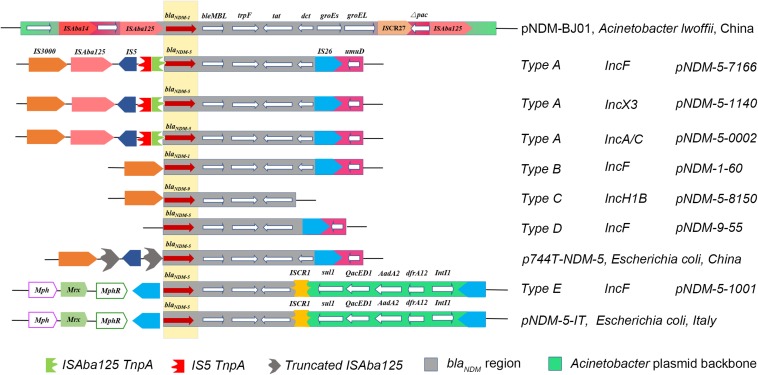
Comparison of the genetic elements surrounding the *bla*_NDM__–__5_ gene identified in this study with the other similar sequences. Reference sequences: pNDM-BJ01 (GenBank accession No. JQ001791) from an *A. lwoffii* strain, p744T-NDM5 (GenBank accession No. MF547511.1) from an *E. coli* strain, and pNDM-5-IT (GenBank accession No. MG649062.1) from an *E. coli* strain.

For genetic environments, 50 NDM-positive isolates were divided into five different types (A to E) based on the analysis of genetic structures, among which type A was the most common (*n* = 25), followed by type E (*n* = 9), type B (*n* = 8), type D (*n* = 5), and type C (*n* = 3). For type A, the flanking genetic structure of the *bla*_NDM–__5_ gene was composed of an IS*3000* and incomplete IS*Aba125* interrupted by IS5 located upstream, and the genes *ble*_MBL_ (bleomycin resistance gene), *trpF* (phosphoribosylanthranilate isomerase), *tat* (DsbC superfamily protein), *dct*, *IS26*, and *umD*. Type B had a complete deletion of IS*3000* and IS*Aba125;* type C and type D only had *IS3000* and *IS26*, respectively. Type E, another prevalent type which was different from type A, harbored another insertion sequence (ISCR1), followed by the *sul* gene (sulfonamide-resistant dihydropteroate synthase), the *QacED1* (quaternary ammonium compound resistance protein), the *AadA2* (aminoglycoside nucleotidyltransferase), the *dfrA12* (dihydrofolate reductase), and the *IntI1* (class 1 integron integrase) genes downstream, the *MphR* (Macrolide 2′-phosphotransferase), the *Mrx* (major facilitator family protein transporter), and the *Mph* [Mph(A) family macrolide 2′-phosphotransferase] upstream (Figure 3). As for the *bla*_KPC–__2_ gene, we did not extract a KPC-2-expressing plasmid in this isolate. Moreover, multiple transformation and transconjugation experiments by the *bla*_KPC–__2_-carrying bacteria failed. Thus, we speculated that the *bla*_KPC–__2_ gene was most likely to be located on the chromosome.

## Discussion

In the present study, 37.61% (41/109) of the CR-EC isolates were identified to be NDM-5-producers, with most isolates carrying the IncF-type plasmids and ST167 being the most common ST type. Moreover, a clinical carbapenem-resistant *E. coli* isolate co-harboring *bla*_KPC–__2_ and *bla*_NDM–__5_ was reported for the first time, with the characterizations of its genetic environment. Our findings revealed a new potential threat of NDM-5-postive CR-EC in mainland China, emphasizing an urgent need to control their further spread.

Some conclusions of this study were noteworthy.

First, our results revealed that the main carbapenem resistance mechanism of the CR-EC isolates collected in our hospital could be attributed to the productions of carbapenemases and ESBL/AmpC enzymes combined with porin deficiencies. Notably, while NDM-1 subtype was reported to be the most prevalent from previous studies in China (Tuem et al., 2018; Markovska et al., 2019), NDM-5 was observed to be prevalent among the CR-EC isolates. Most importantly, our study revealed that the most prevalent epidemic incompatibility type of the *bla*_NDM–__5_-carrying plasmids in our region is IncF (such as IncFIA, IncFIB, and IncFII), which was substantially different from those reported by the other NDM-5 studies (IncA/C and IncX3-type) (Huang et al., 2016; Li et al., 2018), indicating that a new molecular epidemiological CR-EC will be prevalent in China. In addition, a more recent study from eastern China have shown several *bla*_NDM–__5_-positive *E. coli* infections linked to ST167-type CR-EC (Sun et al., 2019), which was also located on the IncX3 plasmid. Moreover, comparisions of the susceptibility results revealed that the *E. coli* isolates carrying the *bla*_NDM–__5_ gene were more resistant than those with *bla*_NDM–__1_ or *bla*_NDM–__9_.

Second, we reported for the first time a clinical *E. coli* strain co-producing NDM-5 and KPC-2, which was isolated from sputum of a patient with respiratory infection. This isolate showed high-level resistances to many antibiotics, but remained susceptible to both tigecycline and colistin. Moreover, while the *bla*_NDM–__5_ gene was demonstrated to be located on separate IncF-type plasmids, the *bla*_KPC–__2_ gene failed to transfer into a recipient isolate with repeated experiments. To further determine the location of the *bla*_KPC–__2_ gene, we performed high-throughput plasmid sequencing and did not find the *bla*_KPC–__2_ gene in plasmid sequencing results, indicating that the *bla*_KPC–__2_ gene might be located on the chromosome.

Third, we found five different types of *bla*_NDM_ gene environments. Of the greatest interest was that all the NDM-producers all carried the highly conserved regions (*bla*_NDM_-*ble*_MBL_-*trpF*-*tat*) surrounding the *bla*_NDM_ gene, suggesting that these four genes were important elements that produced resistances and were not lost during NDM-5 transmission. To the best of our knowledge, this is also the first report of Type C in China, which showed a similar genetic context to pNDM-5-IT from Italy. However, none of these patients in our study had a history of traveling abroad. As previous researches have demonstrated that the rolling circle replication of *ISCR1* element could produce the upstream adjacent antibiotic resistance genes, which could be rescued through recombination between homologous fragments (Li et al., 2014; Cheng et al., 2016), we speculated that the *ISCR1* element in type C could be an important gene assisting *bla*_NDM_ spread via rolling circle transposition.

## Conclusion

In summary, the present study revealed a new emergence of NDM-5-producing CR-EC in a teaching hospital in Chongqing, China, described the first report of a *bla*_KPC–__2_ and *bla*_NDM–__5_-coharboring *E. coli* isolate, and indicated for the first time that the IncF-type plasmids may contribute to the prevalence of *bla*_NDM–__5_, highlighting an urgent need to develop effective measures to prevent and control the further spread of *bla*_NDM–__5_ in China.

## Data Availability Statement

The sequences described in this paper have been submitted to GenBank with the following accession numbers: No. MH985166 (*Escherichia coli* p-NDM-5-1140), MH985167 (*Escherichia coli* p-NDM-5-1001), MH985168 (*Escherichia coli* p-NDM-5-7166), MH985169 (*Escherichia coli* p-NDM-5-8150), MH985170 (*Escherichia coli* p-NDM-9-55), and MH985171 (*Escherichia coli* p-NDM-1-60).

## Author Contributions

SH and HZ designed the study. HZ, XJ, SL, HL, and XW performed the experiments. HZ and XJ analyzed the data. HZ, XJ, and SH wrote this manuscript.

## Conflict of Interest

The authors declare that the research was conducted in the absence of any commercial or financial relationships that could be construed as a potential conflict of interest.

## Footnotes

1 https://sourceforge.net/projects/soapdenovo2/

2 http://www.ncbi.nlm.nih.gov/gorf/gorf.html

3 http://blast.ncbi.nlm.nih.gov/Blast.cgi

4 http://topaz.gatech.edu/GeneMark/

5 https://www.snapgene.com/

6 https://sourceforge.net/projects/mauve/

7 http://mlst.warwick.ac.uk/mlst/dbs/Ecoli
