# Supplementary material for: Emergence of NDM-5-Producing Escherichia coli in a Teaching Hospital in Chongqing, China: IncF-Type Plasmids May Contribute to the Prevalence of blaNDM–5
Source: Front Microbiol. 2020 Mar 6;11:334. doi: 10.3389/fmicb.2020.00334 (PMC7069339; doi:10.3389/fmicb.2020.00334)
Supplement: Supplementary file 1 [file Table_1.DOCX]

**Supplementary Table S1 Primers Used for the Screening of the carbapenemases, ESBLs, AmpC and Porin genes in CR-EC Isolates**

|  | Genes | primer（5’-3’） | [annealing](javascript:;) [temperature](javascript:;)  (℃) | [annealing](javascript:;) [time](javascript:;)  (℃) | Length (bases) | References |
| --- | --- | --- | --- | --- | --- | --- |
| carbapenemase  genes | *bla*_KPC_ | F CATTCAAGGGCTTTCTTGCTGC  R ACGACGGCATAGTCATTTGC | 55 | 45s | 538 | (Dallenne et al., 2010) |
|  | *bla*_IMP_ | F CATGGTTTGGTTGTTCTTGT  R ATAATTTAGCGGACTTTGGC | 55 | 45s | 488 | NA |
|  | *bla*_VIM_ | F TTATGGAGCAGCAACGATGT  R CAAAAGTCCCGCTCCAACGA | 52 | 40s | 920 | NA |
|  | *bla*_NDM_ | F CGGAATGGCTCATCACGATC  R GGTTTGGCGATCTGGTTTTC | 55 | 60s | 621 | (Krishnaraju et al., 2015) |
|  | *bla*_OXA-48_ | F GCTTGATCGCCCTCGATT  R GATTTGCTCCGTGGCCGAAA | 57 | 40s | 281 | (Woodford et al., 2006) |
|  | *bla*OXA-23-like | F GATCGGATTGGAGAACCAGA  R ATTTCTGACCGCATTTCCAT | 52 | 40s | 501 | (Woodford et al., 2006) |
|  | *bla*OXA-24-like | F GGTTAGTTGGCCCCCTTAAA  R AGTTGAGCGAAAAGGGGATT | 52 | 40s | 246 | (Woodford et al., 2006) |
|  | *bla*OXA-58-like | F AAGTATTGGGGCTTGTGCTG  R CCCCTCTGCGCTCTACATAC | 52 | 40s | 599 | (Woodford et al., 2006) |
| ESBLs  genes | *bla*CTX-M-15-like | F TTAGGAAGTGTGCCGCTGCA  R CGATATCGTTGGTGGTRCCAT | 60 | 45s | 700 | (Zhang et al., 2014) |
|  | *bla*_CTX-M-1_ | F AAGACTGGGTGTGGCATTGA  R AGGCTGGGTGAAGTAAGTGA | 55 | 45s | 670 | (Zhang et al., 2014) |
|  | *bla*_CTX-M-9_ | F GCTTTATGCGCAGACGAGTG  R GCCAGATCACCGCAATATCA | 55 | 45s | 686 | (Zhang et al., 2014) |
|  | *bla*_SHV_ | F CTTTACTCGCCTTTATCGGC  R TTACCGACCGGCATCTTTCC | 60 | 45s | 1031 | (Zhang et al., 2014) |
|  | *bla*_TEM_ | F CATTTCCGTGTCGCCCTTATTC  R CGTTCATCCATAGTTGCCTGAC | 60 | 60s | 713 | (Dallenne et al., 2010) |
| AmpC  genes | *bla*_ACC_ | F CACCTCCAGCGACTTGTTAC  R GTTAGCCAGCATCACGATCC | 58 | 40s | 346 | (Dallenne et al., 2010) |
|  | *bla*_FOX_ | F CTACAGTGCGGGTGGTTT  R CTATTTGCGGCCAGGTGA | 58 | 40s | 200 | (Dallenne et al., 2010) |
|  | *bla*_MOX_ | F GCAACAACGACAATCCATCCT  R GGGATAGGCGTAACTCTCCCAA | 58 | 40s | 900 | (Dallenne et al., 2010) |
|  | *bla*_DHA_ | F TGATGGCACAGCAGGATATTC  R GCTTTGACTCTTTCGGTATTCG | 58 | 40s | 1000 | (Dallenne et al., 2010) |
|  | *bla*_CIT_ | F CGAAGAGGCAATGACCAGAC  R ACGGACAGGGTTAGGATAGC | 58 | 40s | 600 | (Dallenne et al., 2010) |
|  | *bla*_EBC_ | F CGGTAAAGCCGATGTTGCG  R AGCCTAACCCCTGATACA | 58 | 40s | 700 | (Dallenne et al., 2010) |
| Porin genes | *ompC* | F GAGAATGGACTTGCCGACTG  R CGAACGGTCGCAAGAGTA | 55 | 55s | 1289 | NA |
|  | *ompF* | F CAGAACTTATTGACGGCAG  R CGGGACGTTCATCGGCAC | 55 | 55s | 1410 | NA |

NA: not applicable.

References:

Dallenne, C., Da Costa, A., Decre, D., Favier, C., and Arlet, G. (2010). Development of a set of multiplex PCR assays for the detection of genes encoding important beta-lactamases in Enterobacteriaceae. *J Antimicrob Chemother* 65**,** 490-495.

Krishnaraju, M., Kamatchi, C., Jha, A.K., Devasena, N., Vennila, R., Sumathi, G., and Vaidyanathan, R. (2015). Complete sequencing of an IncX3 plasmid carrying blaNDM-5 allele reveals an early stage in the dissemination of the blaNDM gene. *Indian J Med Microbiol* 33**,** 30-38.

Woodford, N., Ellington, M.J., Coelho, J.M., Turton, J.F., Ward, M.E., Brown, S., Amyes, S.G., and Livermore, D.M. (2006). Multiplex PCR for genes encoding prevalent OXA carbapenemases in Acinetobacter spp. *Int J Antimicrob Agents* 27**,** 351-353.

Zhang, C., Xu, X., Pu, S., Huang, S., Sun, J., Yang, S., and Zhang, L. (2014). Characterization of carbapenemases, extended spectrum beta-lactamases, quinolone resistance and aminoglycoside resistance determinants in carbapenem-non-susceptible Escherichia coli from a teaching hospital in Chongqing, Southwest China. *Infect Genet Evol* 27**,** 271-276.
