# Supplementary material for: Emergence of NDM-5-Producing Escherichia coli in a Teaching Hospital in Chongqing, China: IncF-Type Plasmids May Contribute to the Prevalence of blaNDM–5
Source: Front Microbiol. 2020 Mar 6;11:334. doi: 10.3389/fmicb.2020.00334 (PMC7069339; doi:10.3389/fmicb.2020.00334)
Supplement: Supplementary file 2 [file Table_2.DOCX]

**Table S2 The primers for the sequencing of the genetic environment surrounding *bla*_NDM_ (part 1)**

| Primer | Sequence(5'–3') | Product length (bp) |
| --- | --- | --- |
| For5198 | GCTCATAGTAGATTTTGGGGG | 1065 |
| Re6262 | GGAGTTCGTAGGGATGCAGTT |  |
| For6154 | TGCAGCTCCATCAGCAAAAGG | 1177 |
| Re7330 | TCGGGTGAAGTCGGGAAAATC |  |
| For6978 | GCCCCTATTCTCTCGGCTTTC | 1285 |
| Re8262 | TTCTTCCCCTATCCTGACCTC |  |
| For7924 | GGCGACGCTGGATAGAACA | 939 |
| Re8862 | ACTCACGCGCATCAGGACA |  |
| For8568 | ACTTGGCCTTGCTGTCCTT | 1363 |
| Re9930 | GCGTTGCTGCTCTTTGTTC |  |
| For9514 | GAGATTTTCTTGTCCCGCA | 1081 |
| Re10594 | TTGGCTTACACCATTAGGG |  |
| For10318 | AAAGCCAGATACAAGGGGT | 935 |
| Re11252 | CTCAGCAAATAGCAAAGCG |  |
| For11016 | TTTCTTGAACTTCGGCTGG | 1001 |
| Re12016 | GCTATGGAATGAGTGCGCT |  |
| For11882 | GGGGATGCCTGTTTGACGA | 1147 |
| Re13028 | CGGGGATTGCGGATGTACT |  |
| For12660 | AACGGGTTTTGAAGGTCTC | 1169 |
| Re13828 | AGCGGTGTTTAAGGATTGG |  |
| For13486 | GCCGTTGAGTTCGTTTTGT | 1343 |
| Re14828 | CCCTATTTGGGCTCTGTTT |  |
| NDM-F | GGTTTGGCGATCTGGTTTTC | 621^11^ |
| NDM-R | CGGAATGGCTCATCACGATC |  |
