# Supplementary material for: Emergence of NDM-5-Producing Escherichia coli in a Teaching Hospital in Chongqing, China: IncF-Type Plasmids May Contribute to the Prevalence of blaNDM–5
Source: Front Microbiol. 2020 Mar 6;11:334. doi: 10.3389/fmicb.2020.00334 (PMC7069339; doi:10.3389/fmicb.2020.00334)
Supplement: Supplementary file 3 [file Table_3.DOCX]

**Table S3 The primers for the sequencing of the genetic environment surrounding *bla*_NDM-5_ (part 2)**

| Primer | Sequence(5'–3') | Product length (bp) |
| --- | --- | --- |
| IS26-F | CTTATCATCCCCTTTTGC | 704 |
| IS26-R | GCAGCCTTTTGTCTTATTCA |  |
| MphR(A)-F | TGTGCCTGGAGGAGTTGG | 584 |
| MphR(A)-F | GCTCGCTTCTGCTTTCACA |  |
| TrpF-F | CGTTAGATTTTCCCGACTTCAC | 365 |
| TrpF-R | ATGCCCGCGAAAATCAAG |  |
| DsbD -F | TCCGGCCTGCCCGAATACCT | 1031 |
| DsbD -R | CTCGGGTGAAGTCGGGAAA |  |
| ISCR1 -F | TGACCGTGAAGTACCAGG | 1541 |
| ISCR1 -R | CGAGACCTTCCGATTTGT |  |
| ISAba125 | A TGT ATA TTT CTG TGA CCC AC | 1356 |
| ISAba125 | ACA CCA TTA GAG AAA TTT GC |  |
| bleo-Rev | GGC GAT GAC AGC ATC ATC CG | 365 |
